# Radiometric and safety assessment of a ‘low‐level red‐light’ myopia control device

**DOI:** 10.1111/opo.70009

**Published:** 2025-09-04

**Authors:** Josh Richards, Jennifer J. Hunter, Javier Gantes‐Nuñez, Arthur Bradley, Pete Kollbaum

**Affiliations:** ^1^ School of Optometry Indiana University Bloomington Indiana USA; ^2^ School of Optometry and Vision Science University of Waterloo Waterloo Ontario Canada; ^3^ Midwestern University Arizona College of Optometry Glendale Arizona USA; ^4^ CooperVision, Inc. San Ramon California USA

**Keywords:** low‐level red‐light therapy, myopia control, photobiomodulation therapy, retinal hazard

## Abstract

**Purpose:**

Recent literature has demonstrated that ‘low‐level red‐light therapy’ may be effective at slowing axial elongation in children, but reports have questioned the safety of some red‐light devices. The current work explored the beam characteristics and hazard potential for a myopia control red‐light device.

**Methods:**

The optical design and exiting beam characteristics of a sample red‐light myopia control device (SECONEE sky‐n1201a) were quantified by measuring beam divergence and total flux passing through artificial pupils of 1–9 mm diameter placed at the corneal plane. Retinal exposure levels were compared to American National Standards Institute (ANSI) limits for ophthalmic instruments (Z80.36‐2021).

**Results:**

Two 655 nm laser diodes created exiting red‐light beams (~9 mm in diameter) that were approximately collimated by an internal pair of +10.00 D eye‐piece lenses resulting in exit vergences ranging from −3.25 to −7.75 D as interpupillary distance settings were adjusted from 52 to 70 mm. Radiant power (mW) passing through the artificial pupils increased from 0.005 to 0.65 mW as pupil diameters increased from 1 to 9 mm. Using ANSI Z80.36‐2021, time‐averaged retinal radiant exposures (J/cm^2^) exceeded the *thermal* and *photochemical* limits for pupil diameters >1 mm and >5 mm, respectively. The time to reach potential *photochemical* radiation hazard (*t*_max_) was less than 100 s for pupil diameters >5 mm.

**Conclusions:**

The optical design and resulting beam characteristics of this sample red‐light myopia control device generate retinal exposure levels that vary with pupil diameter, accommodation and eye movements. Conservative estimates reveal retinal radiant exposures that can exceed ANSI safety limits. The ‘low‐level’ moniker is inappropriate for a device that either exceeds or is just below the ANSI threshold for potential retinal hazard.

**Key points**

- The optical design of this sample red‐light myopia control device created retinal exposure levels that varied with pupil diameter, accommodation and eye movements.
- Conservative estimates of retinal energy densities exceeded American National Standards Institute hazard limits.
- Eye care professionals should exercise caution when prescribing the use of instruments utilising high intensity light sources for their patients.

## INTRODUCTION

In response to the current and predicted future prevalence levels of myopia and high myopia,^1^ (e.g., myopia prevalence currently >80% in parts of East Asia)^2^, ^3^ several treatment strategies designed to slow or stop the progression of myopia have been introduced into clinical eye‐care practice.^4^ Experimental studies indicate that retinal mechanisms control eye growth,^5^ and characteristics of the retinal image including light level,^6^, ^7^ defocus,^5^ contrast^8^ and spectral properties^9^ have been implicated as controlling factors.^10^ All‐day rearing of young (infant, juvenile and adolescent) tree shrews in red‐light induced hyperopic shifts,^9^ which increased with higher exposure levels, increased exposure times^11^ and narrower spectra.^12^ All‐day red‐light rearing prevented both defocus‐induced and form‐deprivation myopia in infant macaques.^13^

Exposure to red light has been implemented as a myopia control method in children. However, unlike the animal studies, red‐light therapy in children restricts exposures to a few minutes per day (e.g., twice daily for 3 min). These short‐term exposures have been shown to reduce myopia progression and axial elongation in myopic children.^14^, ^15^, ^16^ Although the red‐light therapy devices are labelled and described as ‘low‐level’, concerns have been expressed that their light output may exceed safe levels of retinal exposure^17^, ^18^ and one documented case of retinal damage has been reported.^19^ Safety concerns have prompted China's National Medical Products Administration to require a safety assessment before any red‐light devices can be used in clinical trials.^20^, ^21^ These concerns and the general absence of radiometric data for these devices motivated the current study which examined the optical design, radiant characteristics and hazard potential of one such ‘low‐level red‐light’ therapeutic device.

### Device radiometry

**FIGURE 1:**
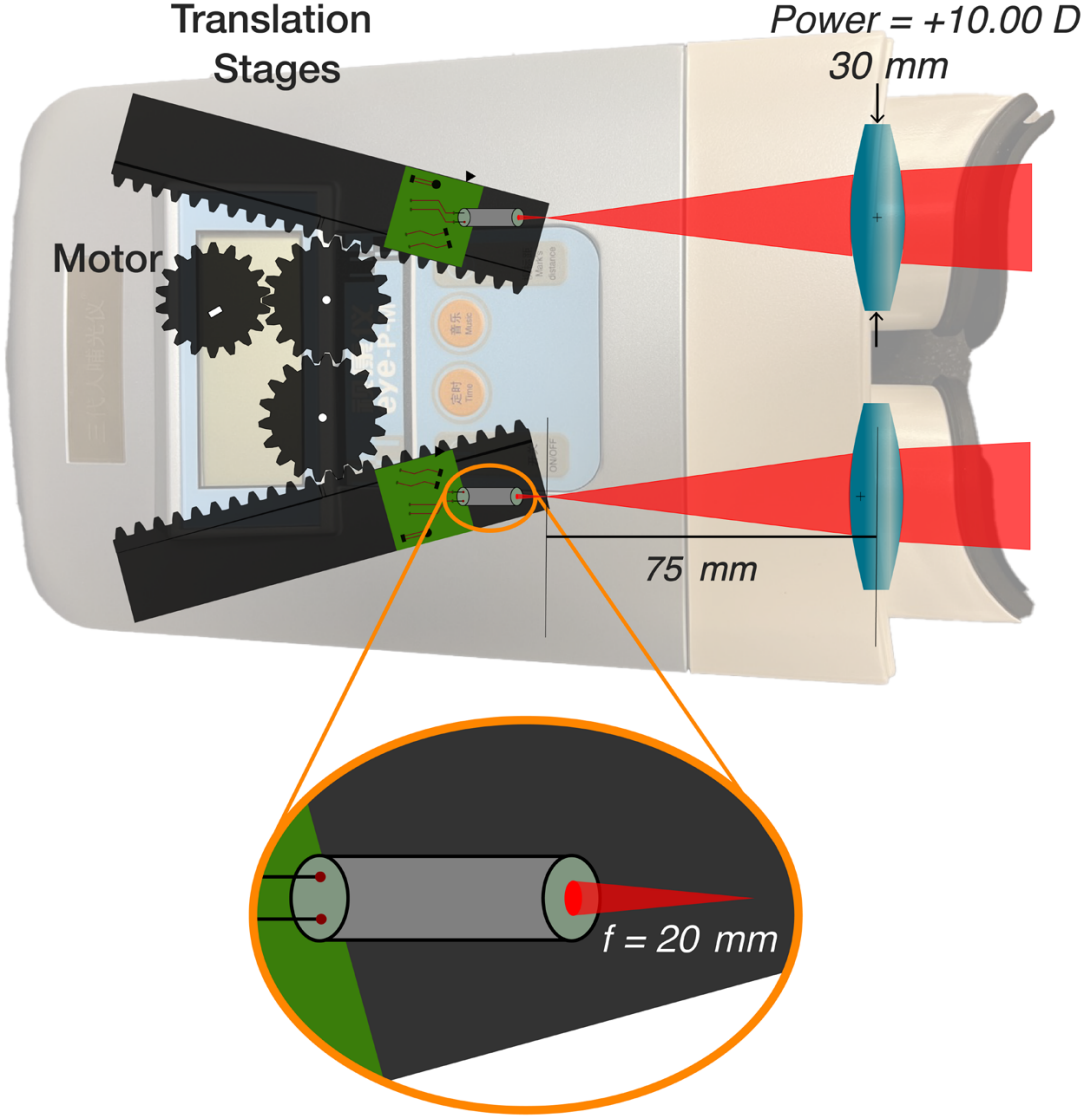
A schematic of the investigated device (SECONEE sky‐n1201a) shows the internal components: A pair of lasers (*f* = 20 mm) mounted to two translation stages 75 mm from the +10.00 D 30 mm diameter eyepiece lenses (for a 70 mm interpupillary distance).

The instrument design and exiting beam characteristics of a sample red‐light myopia control therapeutic device (SECONEE sky‐n1201a, Beijing Mingren Shikang Science & Technology Co., Ltd) were examined. The instrument, which can be either handheld or rested securely on a desk or table, allowed the user to adjust the interpupillary distance (IPD) within a range of 52–70 mm before initiating an automatically timed 3‐min exposure. The device contained a pair of solid‐state lasers oriented parallel to the instrument axis, each mounted on translation stages oriented 20.0° from the instrument axis (Figure 1). Control of the lateral separation of the laser sources was achieved by forward or backward translation along these tracks. The lasers were focused 20 mm from their exit apertures, after which the right and left eye beams diverged towards the pair of eyepiece lenses (+10.00 D, 30 mm diameter). Changes in the lateral separation of the exiting beams were accompanied by axial movement which altered the distance of the laser focus from the eyepiece lenses, thus changing the beam diameters and vergences exiting the device.

**FIGURE 2:**
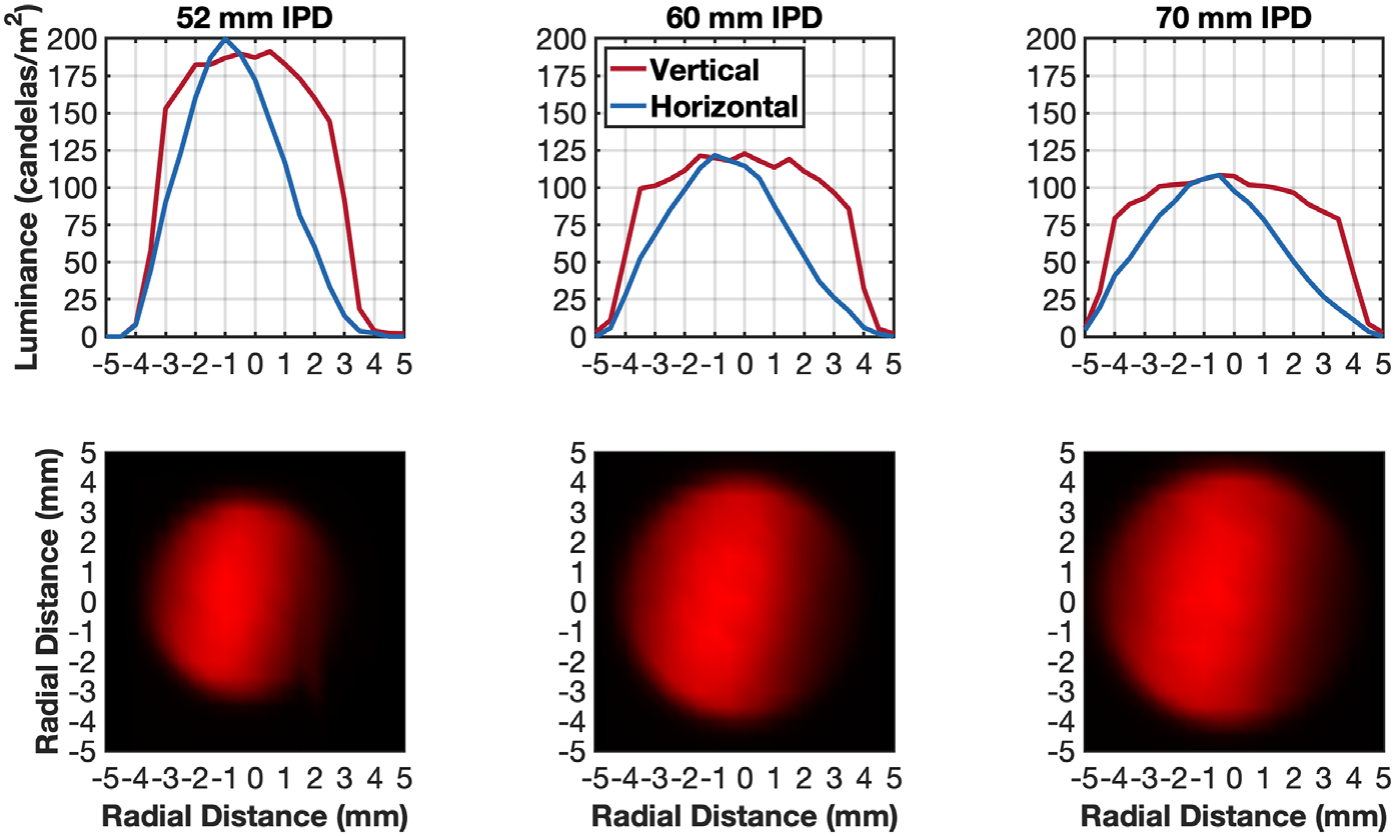
Horizontal and vertical luminance profiles (top row) of the beams at the corneal plane for 52, 60 and 70 mm interpupillary distance (IPD) settings. Luminance maps (bottom row) were calculated by radial interpolation of the horizontal and vertical slices.

When IPD settings were changed from 70 mm to 52 mm, the distance from the focal point of the laser to the centre of the +10.00 D eyepiece lens decreased from 75 mm to 56 mm. This altered the input vergence of the beam at the eyepiece lens from −13.25 to −17.86 D, increasing the exit beam vergence from −3.25 to −7.86 D (measured at the plane of the eye piece lens). As the device lasers were moved towards the eyepiece lenses, the beam size entering and leaving the eyepiece lenses was reduced. The exiting beam spectral characteristics (measured with a GL Spectris 1.0 spectrometer, gloptic.com) revealed a narrow spectrum with a peak at approximately 655 nm. The sizes and spatial profiles of the exiting beams at the nominal corneal plane were quantified by placing a diffusing plate at the corneal plane and measuring the plate luminance using a Photo Research Spotmeter (jadaktech.com/products/photo‐research) which integrated photons over a 0.5 mm diameter circular area and sampled every 0.5 mm across the horizontal and vertical extent of the beam (Figure 2b, top row). Maps of the full beam luminance values (Figure 2b, bottom row) were created by interpolation using cosine and sine weights to the horizontal and vertical slices. As anticipated from the oblique track angles (Figure 1), the smaller IPD setting led to smaller corneal plane beam diameters. The horizontal (H) and vertical (V) beam widths for IPD settings of 70, 60 and 52 mm were 9.0 (H) and 9.2 mm (V), 8.2 (H) and 8.9 mm (V) and 7.3 (H) and 7.8 (V) mm, respectively.

**FIGURE 3:**
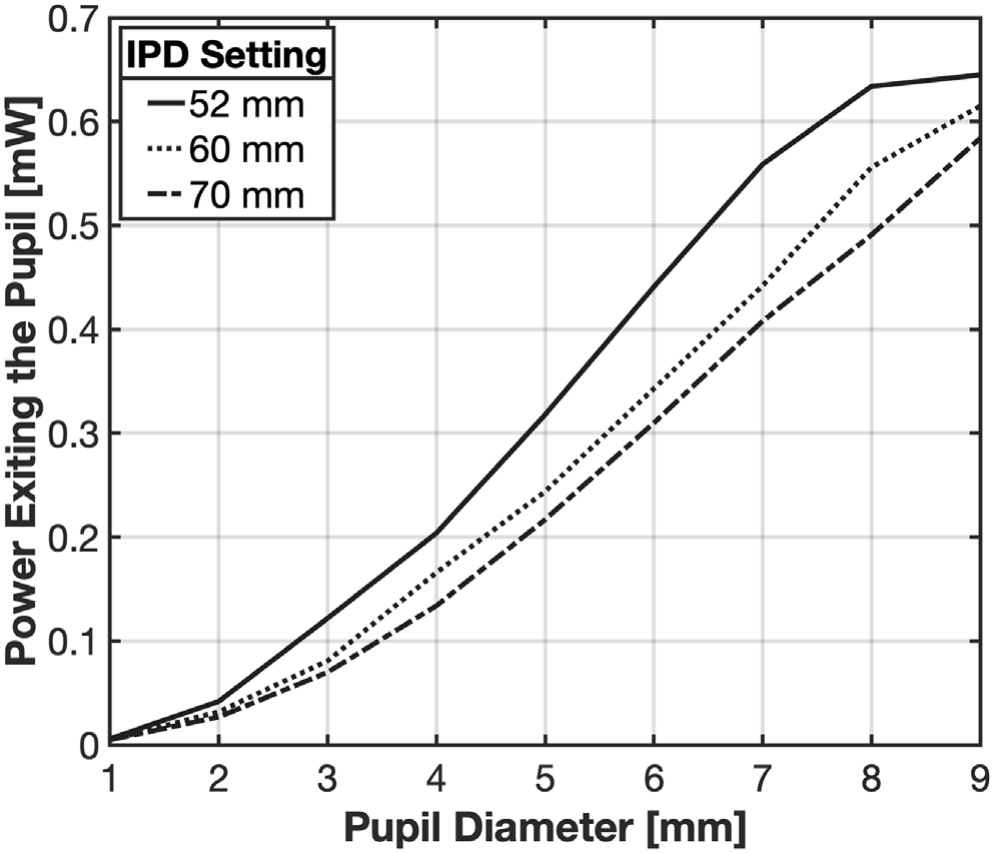
Radiant power exiting artificial pupils placed at the corneal plane with diameters ranging from 1 mm to 9 mm for device interpupillary distance (IPD) settings of 52 mm, 60 mm and 70 mm.

Because of the approximately 9 mm beam diameters at the corneal plane, the patient/user's eye pupil diameter will impact the amount of light entering the eye. Also, because the smaller IPD setting concentrates the beam, children with smaller IPDs will be exposed to higher irradiance than children with larger IPDs with pupil diameters <9 mm. Radiant power passing through 1–9 mm diameter artificial pupils placed in the corneal plane was assessed with a THORLABS Optical Power Meter PM100D (thorlabs.com) (a sensor diameter of 1 cm) placed directly behind the artificial pupils. As pupil diameters increased from 1 mm to 9 mm, radiant power passing through the pupil increased from 0.006 to 0.65, 0.005 to 0.62 and 0.005 to 0.58 mW for IPD settings of 52 mm, 60 mm and 70 mm, respectively (Figure 3). Because of the non‐uniform beam profile and greater radiant power at the beam centre (Figure 2), the radiant power did not scale proportionally with pupil area.

### Device safety

The safety of light sources that irradiate the retina depends on several factors, including radiant power, wavelength, beam characteristics, spatial distribution of light at the retina and both duration and frequency of exposure. The eye safety of lasers is generally assessed using the American National Standards Institute (ANSI) Z136.1‐2022, which is designed to evaluate safety for inadvertent exposure to laser beams. Such inadvertent exposure would often be accompanied by an aversion response—rapid eye movement and lid closure. The ANSI standard used to assess the potential hazard of ‘ophthalmic devices that direct optical radiation into or at the eye’ (ANSI Z80.36‐2021), is directly applicable to the application of red‐light therapy for myopia control for which there is no aversion response and in fact, children are specifically instructed to sustain viewing. These standards define retinal radiant exposures for assessing possible risks to the retina from both *photochemical* and *thermal* damage.

For extended (time‐limited) exposures, the upper retinal exposure limit to avoid *photochemical* hazard is 2.2 J/cm^2^ (ANSI Z80.36‐2021, Table 3). Time‐integrated retinal exposure (retinal radiant exposure) to be compared to the limit is defined as the product of retinal irradiance ($E_{\lambda }$), exposure duration (*t*) and a wavelength dependent weighting factor $A\left(\lambda \right)$ that reflects the relative spectral sensitivity for *photochemical* damage (0.01 for 655 nm). Retinal irradiance ($E_{\lambda }$) is calculated by averaging the highest localised radiant power over a specified retinal area. For assessment of *photochemical* hazard, the standard includes two estimates of irradiated retinal areas, one of which has a diameter of 30 μm for a stabilised image (the immobilised eye case) such as in ocular surgery,^22^ and another which has a diameter of 180 μm for normal (unstabilised) viewing conditions (Z80.36‐2021, section 5.4). As the retinal location of a small point source is moved by eye and head movements, the accumulated retinal exposure at any one location decreases. The larger and smaller circular retinal areas used in Z80.36‐2021 have areas of 2.54 × 10^−4^ and 7.07 × 10^−6^ cm^2^, respectively, differing by a factor of 36. A 36 times increase in area results in a 36 times decrease in calculated $E_{\lambda }$. During treatment with the SECONEE device, the head may move and eye movements are not constrained through eye immobilisation; therefore, for assessment of *photochemical* hazard, retinal irradiance $E_{\lambda }$, is calculated using the larger retinal area. Actual eye movements during therapy are unknown. Because heat dissipates during long exposures, the *thermal* hazard limit in J/cm^2^ increases with stimulus duration (6t^0.75^ J/cm^2^, Z80.36‐2021, Table 3). The spectral weight for the thermal hazard at 655 nm $R\left(\lambda \right)$ is 1.2 and the spatial averaging used for *thermal* hazard calculations employs a circle diameter of 30 μm.^22^

The ANSI Z80.36‐2021 standard defines a device as safe for continuous viewing up to 10,000 s as a *Group 1* device if its retinal radiant exposures do not exceed the upper limits of exposure defined in section 5.4. Specifically, to qualify as a *Group 1* device, the weighted retinal radiant exposure must neither exceed the limits for *photochemical* damage (2.2 J/cm^2^, 5.4.2.3) nor the limits for *thermal* damage (6*t^3/4^ J/cm^2^ = 294.85 J/cm^2^, Z80.36‐2021 5.4.2.4). A time‐limited or pulsed instrument also does not qualify as a Group 1 instrument if it only meets these limits for a single retinal exposure.

Weighted retinal radiant exposures ($H_{A-R}$, Table 5.4.2.3) to be compared to the *photochemical* limit are the product (Equation (1)) of the retinal irradiance ($E_{\lambda }$), exposure time (t) and the wavelength‐dependent weighting factor ($A\left(\lambda \right)$). Therefore, after unweighting by $A\left(\lambda \right)$ (0.01 for a 655 nm source, ANSI Z80.36‐2021, appendix A), the unweighted *photochemical* exposure limit is 220 J/cm^2^.

(1) $$H_{A-R}=t*E_{\lambda }*A\left(\lambda \right)$$

*Thermal* hazard for long exposures is calculated similarly, although using a different wavelength‐dependent weighting factor ($R\left(\lambda \right)$ Equation (2)). After unweighting by $R\left(\lambda \right)$, which is 1.22 for a 655 nm source (Z80.36‐2021, appendix A), the unweighted limit is 242 J/cm^2^.

(2) $$H_{\mathrm{VIR}-R}=t*E_{\lambda }*R\left(\lambda \right)$$

**FIGURE 4:**
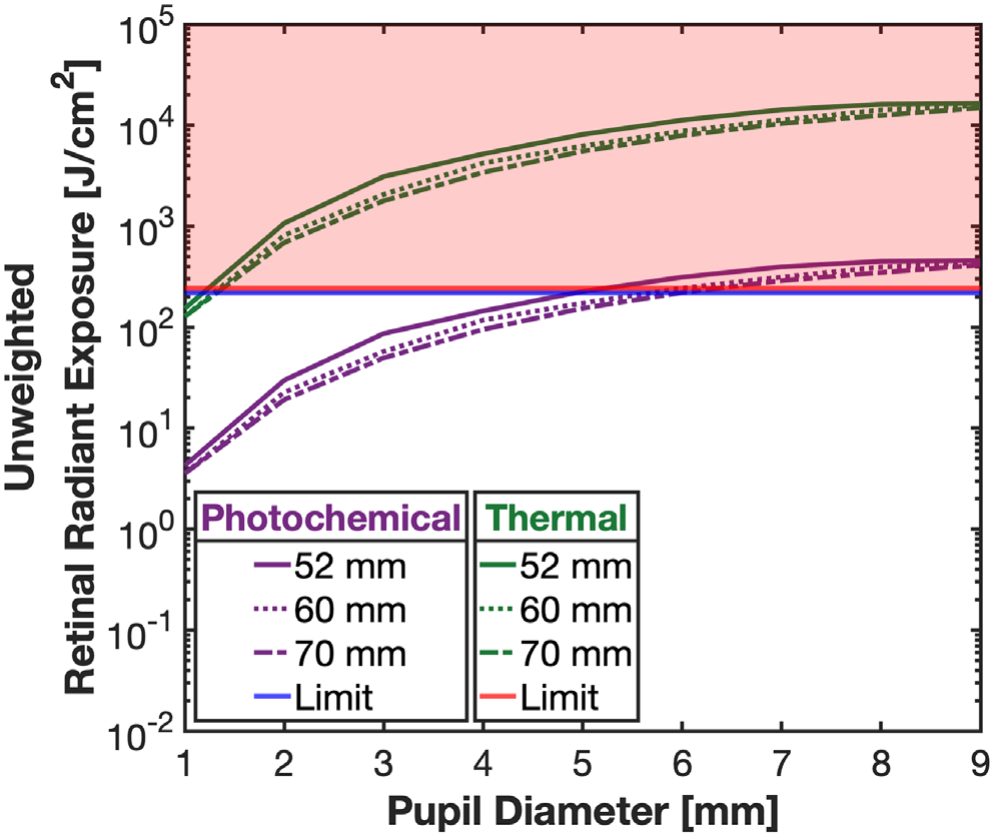
Unweighted retinal radiant exposures for assessment of *photochemical* hazard (purple) and *thermal* hazard (green) for pupil diameters ranging between 1 mm and 9 mm for 52 mm (solid lines), 60 mm (dotted lines), and 70 mm (dashed lines) interpupillary distance (IPD) settings. The retinal *photochemical* and *thermal* unweighted limits (5.4.2, ANSI Z80.36‐2021) are indicated by blue and red horizontal lines, respectively. Exposures highlighted in red exceed retinal hazard limits.

Figure 4 plots the unweighted retinal radiant exposures for evaluation of *photochemical* hazard (purple) and *thermal* hazard (green) as a function of pupil size for a single 180‐s exposure without eye immobilisation. Retinal exposure levels are much higher for the thermal hazard calculation because of the 36 times smaller area over which maximum intensity was averaged. For the smallest to the largest pupils, and an IPD setting of 60 mm, retinal radiant exposure for *photochemical* hazards ranged from 4 to 436 J/cm^2^ and for *thermal* hazards the range was 153 to 16,421 J/cm^2^. For an eye with a 7 mm pupil and an IPD of 60 mm, the unweighted radiant exposures for evaluation of *photochemical* and *thermal* hazard are 313 and 11,253 J/cm^2^, respectively, that is, the *thermal* retinal radiant exposure exceeding the limit by a factor of 46. These results indicate that use of the device is potentially hazardous for single exposures of 180 s and exceeds the limits for *photochemical* and *thermal* hazard necessary to be classified as a *Group 1* device (section 5.4.2).

**FIGURE 5:**
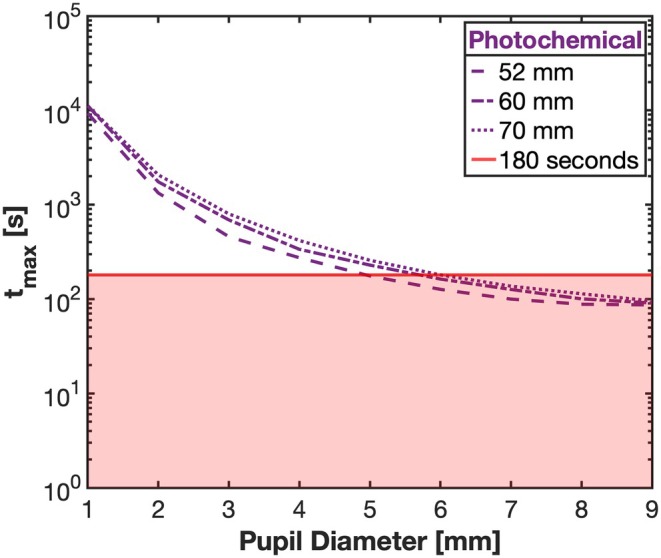
The ‘time to reach a potential optical radiation hazard for photochemical aphakic retinal exposure’ (*t*_max_) for pupil sizes ranging between 1 mm and 9 mm (interpupillary distance (IPD) of 52 mm to 70 mm). *t*_max_ values less than the single exposure time of 180 s (red line) are potentially hazardous.

The ‘time to reach a potential optical radiation hazard for photochemical aphakic retinal exposure’ (*t*_max_) (ANSI Z80.36‐2021 6.5.3) is equal to the time to accumulate an aphakic retinal radiant exposure of 2.2 J/cm^2^ (Equation (3)). Pupil diameter and IPD setting both influenced the maximum safe exposure duration (*t*_max_) which ranged from 11,176 to 86 s (Figure 5). For example, using the device for 180 seconds exceeds the *photochemical* *t*_max_ with pupil diameters greater than approximately 5 mm.

(3) $$t_{\max}=\frac{2.2\;J/{\mathrm{cm}}^{2}}{E_{\lambda }*A_{\lambda }\;\left(W/{\mathrm{cm}}^{2}\right)}$$

## DISCUSSION

The current study examined the radiometric properties of an individual SECONEE sky‐n1201a red‐light myopia control device. The exiting beam power, spectral composition and energy profile were used to evaluate its safety as an ophthalmic instrument using ANSI Z80.36‐2021. The 655 nm red‐light expanded laser beams exiting the instrument were non‐uniform, larger than the eye‐pupil and varied in size and divergence with IPD settings. Safety analysis revealed conservative estimates of retinal energy densities exceeding the Z80.36‐2021 limits to be classified as a *Group 1* device when used for the recommended 180‐s treatment duration. The important role that the pupil plays in regulating retinal irradiance levels with this device highlights the danger of using such a device without knowledge of the child's pupil size.

A recent examination of a similar SECONEE device^17^, ^23^ measured a beam radiant power of 0.2 mW passing through a 7 mm diameter aperture placed 10 cm from the oculars, whereas the current study observed 0.44 mW of beam radiant power measured at the corneal plane. The diverging beam (−5.00 D with IPD set to 60 mm) exiting the device will approximately double its area after propagating 10 cm, thus reducing the beam radiant power by approximately 50%. The results for the 7 mm pupils are, therefore, consistent between the two studies indicating consistent device beam characteristics.

Clinical studies examining the efficacy of SECONEE device have described the device as ‘safe’.^15^, ^24^ However, these same studies also note participant‐reported temporary vision loss of over 2 min associated with exposure to the device. Exposures below recommended levels may still result in retinal damage^25^, ^26^ and short‐term vision loss.^27^ While patients are instructed to use this device twice daily for 3 min, the device does not limit the number of treatments. The cumulative effects of repeated light exposure are reflected in the Z80.36‐2021 which specifies that ‘a time‐limited or pulsed instrument does not quality as a Group 1 instrument if it only meets this limit [retinal photochemical aphakic light hazard] for a single retinal exposure’ within the visible range. Also, the ANSI Z136.1‐2022 standard recommends that for visible light exposure in the presence of eye/head immobilisation, ‘cumulative retinal radiant exposure to overlapping retinal areas shall not exceed [photochemical] limits within 48 hours’. Both standards emphasise the increased risk of repeated exposures, which is a key feature of red‐light myopia therapy (e.g., twice daily).

The 8–9 mm beam diameter at the corneal plane (Figure 2) and low negative beam divergence (Figure 1) means that retinal flux densities will depend upon both pupil size and the accommodative behaviour of the patient. In low light, children's pupil diameters can exceed 7 mm, whereas in the presence of bright fixated lights, pupil diameters can shrink to approximately 3 mm.^28^, ^29^ If a mydriatic has been used (e.g., in the eye examination or as part of atropine therapy for myopia control),^30^ the pupil diameter can remain large, even in the presence of bright light.^31^ The ANSI Z136.1‐2022 guidelines for the safe use of lasers acknowledge the additional risks with pharmacologically dilated pupils and/or eye/head immobilisation and recommend reducing exposure levels under specific conditions (ANSI Z136.1‐2022, section 8.3). The spatial spread of the energy at the retina will also depend upon eye optical magnification, eye movements and any uncorrected refractive error.^22^, ^32^, ^33^ The safety calculations used to generate Figures 4 and 5 assume that the user of the device focused the beam onto the retina but was freely moving their eyes during the treatment protocol. However, for a single 180‐s treatment session and constrained eye movements, the *photochemical* aphakic retinal exposure could exceed safety limits with pupil diameters smaller than 5.5 mm. For example, with complete immobilisation and a focused beam, a single 180‐s exposure will be potentially hazardous with pupils greater than approximately 1.5 mm. Device safety, therefore, may be enhanced with instructions for the user to employ random eye motion during treatment and avoid careful fixation on the light source.

Unlike animals raised in red‐light environments,^9^, ^11^, ^13^ children being treated with red light are alternately exposed to narrowband red (for 3 min) and broad‐spectrum environmental light for multiple hours between treatments.^14^, ^34^, ^35^ Experimental studies in tree shrews showed that alternating equal‐luminance red and white LEDs every 2 s produced greater hyperopic shifts than constant red light.^12^ Finally, the area of the retina exposed to red light differs significantly with the animal studies, which expose the full retina versus the red‐light therapy devices that illuminate a small foveal retinal area. The notion that a small foveally fixated light can regulate eye growth globally is challenged by data showing that local stimulation results in local eye growth in chicks^36^, ^37^, ^38^ and macaques,^39^ as well as experimental evidence showing that foveal function is not required for eye growth regulation.^40^

The tested red‐light myopia control device may be hazardous to the retina (ANSI Z80.36‐2021) for a prescribed single 180‐s duration, despite being identified as a ‘low‐level’ device.^15^ Importantly, however, the safety limits embodied in all currently available ANSI standards were not specifically established by exposing the eyes of children repeatedly to high‐intensity red lights over multiple years, and therefore applications of the ANSI Z80.36‐2021 safety limits may be inappropriate for the specific clinical scenario proposed for these red‐light devices. Careful verification of the safety of red‐light devices before undergoing clinical trials is important, as recent regulatory changes in China have emphasised.^20^, ^21^ Design changes and fixation instructions that spread the energy over larger retinal areas will increase device safety.

## AUTHOR CONTRIBUTIONS

**Josh Richards:** Conceptualization (equal); formal analysis (equal); investigation (equal); methodology (equal); validation (equal); visualization (equal); writing – original draft (equal); writing – review and editing (equal). **Jennifer J. Hunter:** Conceptualization (equal); formal analysis (equal); investigation (equal); methodology (equal); validation (equal); visualization (equal); writing – original draft (equal); writing – review and editing (equal). **Javier Gantes‐Nuñez:** Conceptualization (equal); data curation (equal); investigation (equal); methodology (equal); validation (equal); visualization (equal); writing – original draft (equal); writing – review and editing (equal). **Arthur Bradley:** Conceptualization (equal); formal analysis (equal); investigation (equal); methodology (equal); validation (equal); visualization (equal); writing – original draft (equal); writing – review and editing (equal). **Pete Kollbaum:** Conceptualization (equal); formal analysis (equal); funding acquisition (equal); investigation (equal); methodology (equal); project administration (equal); resources (equal); supervision (equal); validation (equal); visualization (equal); writing – original draft (equal); writing – review and editing (equal).

## CONFLICT OF INTEREST STATEMENT

PK: research: Alcon, CooperVision, EssilorLuxottica, Hoya, Johnson and Johnson Vision and SightGlass Vision; PK: consultant EssilorLuxottica and SightGlass Vision; JH: patent: University of Rochester; AB: CooperVision Inc. employee.
